# Effects of Phenanthrene Exposure on the B-esterases Activities of *Octopus maya* (Voss and Solís Ramírez, 1996) Embryos

**DOI:** 10.1007/s00128-023-03706-8

**Published:** 2023-03-14

**Authors:** Letícia Aguilar, Gissela Moreno-Ortiz, Claudia Caamal-Monsreal, Carlos Rosas, Elsa Noreña-Barroso, María Concepción Gómez-Maldonado, Gabriela Rodríguez-Fuentes

**Affiliations:** 1 Unidad de Química en Sisal, Facultad de Química, Universidad Nacional Autónoma de México, Puerto de Abrigo S/N, Sisal, Yucatán Mexico; 2 Unidad Multidisciplinaria de Docencia e Investigación, Facultad de Ciencias, Universidad Nacional Autónoma de México, Puerto de Abrigo S/N, Sisal, Yucatán Mexico; 3 Unidad de Química en Sisal, Facultad de Química, UNAM, Av. Colón # 503 F X 62 y Reforma Colonia Centro, 97000 Mérida, Yucatán Mexico

**Keywords:** Octopus, Phenantrene, B-esterase

## Abstract

No ecotoxicological information exists on phenanthrene (Phe) exposure in cephalopods, animals of commercial and ecological importance. This study investigated the effect of Phe on two B-esterases, Acetylcholinesterase (AChE) and Carboxylesterases (CbE), in *Octopus maya* embryos. Octopus embryos were exposed to different treatments: control (seawater), solvent control (seawater and DMSO 0.01%), 10 and 100 µg/L of Phe. AChE and CbE activities were measured at different developmental stages (blastula, organogenesis, and growth). B-esterase activities increased in control and solvent control as the embryos developed, showing no statistically significant differences between them. On the other hand, the embryos exposed to Phe had significant differences from controls, and between the high and low concentrations. Our results indicate that B-esterases are sensitive biomarkers of exposure to Phe in *O. maya*. Still, complementary studies are needed to unravel the toxicodynamics of Phe and the implications of the found inhibitory effect in hatched organisms.

*Octopus maya* (Voss and Solís-Ramírez 1966) is endemic to southeastern Mexico and is distributed from Ciudad del Carmen and Bahía de Campeche to Isla Mujeres in the northwest. It is one of Mexico's most important fishery resources (Pascual et al. 2020). From 1998 to 2016, national catches ranged between 9000 and 35,000 t/year, representing 10% of the world's octopus catch (Markaida and Gilly 2016). The high demand for octopus consumption worldwide has led to an increase in demand; world catches increased by about 700% between 1950 and 2015, leading to increased exports of *O. maya* and *O. vulgaris* from Mexico to Asia and Europe (Coronado et al. 2020; Sauer et al. 2021). Despite the large consumption of these animals, there is a lack of information on how they interact with the pollutants in the aquatic environment (Isaacs et al. 1998). Octopuses occupy more than one position in the food chain (Markaida and Gilly 2016), which gives them ecological relevance in aquatic ecosystems. These animals can be directly affected by contaminants, bioaccumulation, and biomagnification processes (Tran et al. 2019). Given the high consumption of octopus worldwide, the lack of knowledge about the effects of pollutants on these animals can significantly impact human health and ecosystems (Bhagat et al. 2016).

Among the aquatic pollutants, polycyclic aromatic hydrocarbons (PAHs) are an important class of persistent organic pollutants found ubiquitously in the environment (Fu et al. 2011). Due to their persistence and potential harmful impact on the ecosystem and human health, the United States Environmental Protection Agency (USEPA) has classified 16 PAHs as priority pollutants (Fu et al. 2011), among which is phenanthrene (Phe) (Chen et al. 2018). Phe is the main component of the total content of PAHs compounds in aquatic habitats (Chen et al. 2018). It is one of the 129 contaminants of most significant concern by the USEPA due to its wide distribution and high toxicity (de Campos et al. 2018). In the coastal waters of the Gulf of Mexico, where *O. maya* is distributed, Narciso-Ortiz et al. (2020) have reported an environmental range of Phe between 7 × 10^–3^ and 1540 µg/L. Due to its relatively low molecular weight, Phe can dissolve in water. It becomes readily bioavailable (Hannam et al. 2010) and is adsorbed onto particles or lipids, exhibiting a high bioaccumulation factor in aquatic organisms (Chen et al. 2018). Several studies performed with invertebrates and fish reported that exposure to crude oil, PAHs, and derivatives cause inhibition of cholinesterases (ChE) activity, including Acetylcholinesterases (AChE, Aguilar et al. 2022; Vieira et al. 2008; Baršiene et al. 2006; Rodríguez-Fuentes et al. 2016).

ChE inhibition could be caused by the action of reactive oxygen species (ROS) that can activate or deactivate ChE (Rico et al. 2007; Rodríguez-Fuentes et al. 2015). It is known that Phe and its metabolites comprise the increase in the production of ROS (Turja et al. 2020). Other enzymes that can provide information on the toxicological level of cells are carboxylesterases (CbE). CbE and AChE are hydrolases classified as B-esterases, as they are inhibited by organophosphate compounds (Laguerre et al. 2009). The goal of the present study was to use B-esterases (AChE and CbE) in *O. maya* embryos exposed to environmentally relevant concentrations of Phe as biomarkers. We hypothesize that the exposure inhibits esterase activities in a concentration-dependent manner.

## Materials and Methods

This study followed the protocols for maintenance, manipulation, and sacrifice of the experimental animals according to certified criteria established by the Institutional Committee for the Care and Use of Laboratory Animals (CICUAL) of the Facultad de Química, UNAM (OFICIO/FQ/CICUAL/461/22). All efforts were made to minimize stress in experimental animals and meet standard levels of animal welfare.

The study was carried out in the Experimental Cephalopod Production Unit (ECPU) at the UMDI-Sisal, Facultad de Ciencias UNAM Yucatán, Mexico. Adult octopuses were caught using artisan lines, with fresh crabs as bait, offshore Sisal (Yucatan, Mexico) at 10–12 m depth. Octopuses were transported in a 120 L circular tank containing local seawater to the laboratory situated 300 m inland.

Adults were acclimated for two weeks in 7 m^3^ aerated tanks supplied with natural seawater (24–26 °C) and fed ad libitum with fresh crab. During that period, the fertilization of the females occurred. After the acclimation, females were placed in 80 L dark tanks with re-circulated seawater at 24 ± 1 °C, 35–37 PSU, pH > 8, and dissolved oxygen > 5 mg/L. Females were maintained under a photoperiod regime to spawn after 25–30 days, i.e., a 10 h light/14 h dark (Rosas et al. 2014), and fed twice a day with fresh crab.

**Fig. 1:**
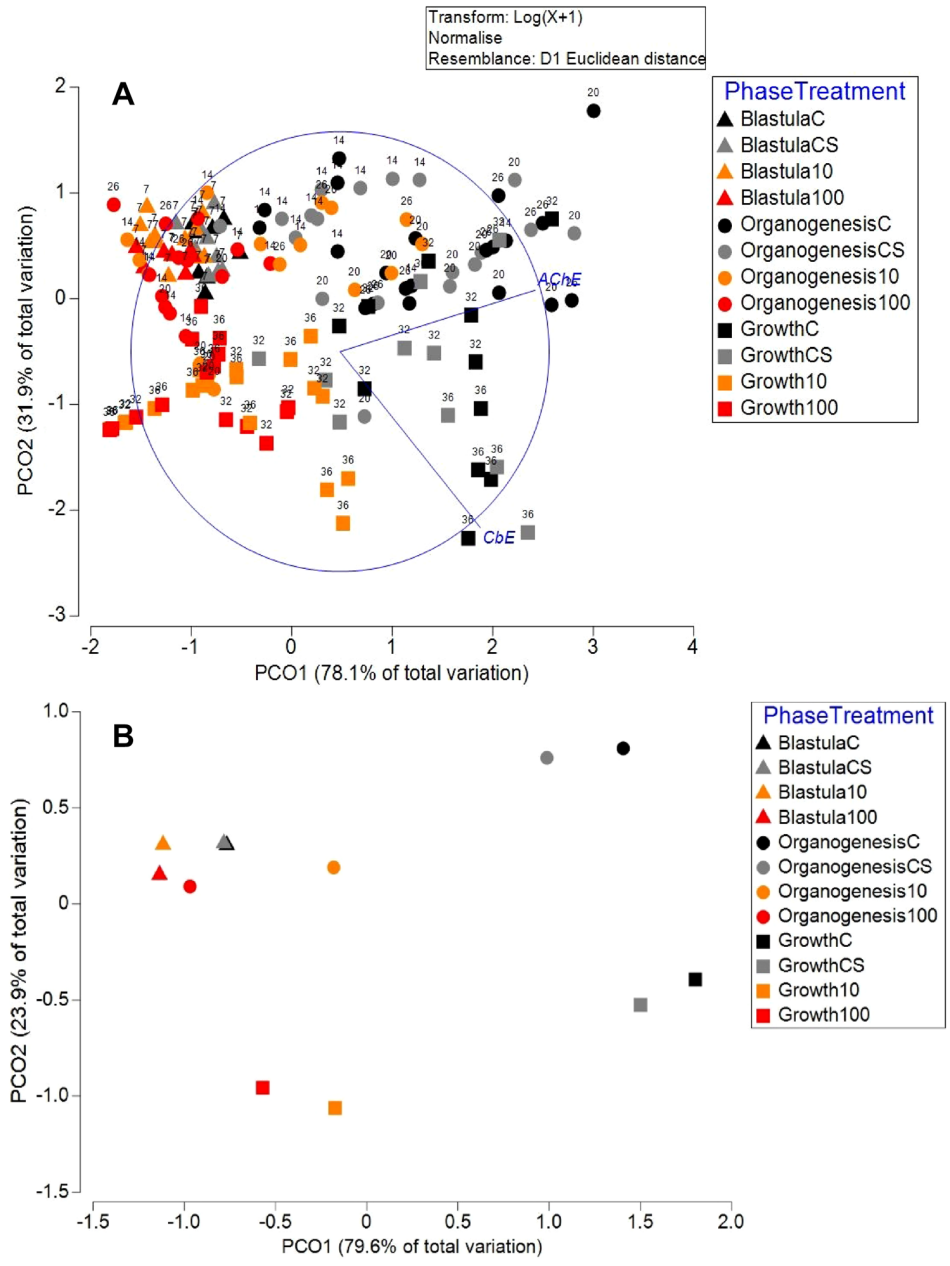
**A** Principal coordinate analysis (PCO) of B-esterases activity in embryos of *O. maya* exposed to 10 and 100 µg/L phenantrene (Phe), control and solvent control (C and CS) at blastula, organogenesis and growth phases. The number over the symbol represent the day sample was taken. **B** Centroids for the combined factors Phase (blastula, organogenesis, and growth) and treatment (control, solvent control, 10 and 100 µg/L Phe)

The experimental design was as follows: 12 days after spawning, 24 clutches of spawned eggs on stages IV-V were transferred to twelve 2 L glass tanks (two clutches per tank, three tanks per treatment) where embryos were exposed to seawater (control), seawater and DMSO 0.01%, 10 and 100 µg/L Phe (Sigma-Aldrich, 98%)). To obtain the nominal Phe concentrations (10 and 100 µg/L), Phe was dissolved in dimethyl sulfoxide (DMSO) and added to filtered seawater (final maximum DMSO concentration of 0.01%) (Lüchmann et al. 2014). Phe concentrations were chosen based on previous reports from bivalves (Hannam et al. 2010; Lüchmann et al. 2011)and realistic ambient concentrations from oil exploration areas (Anyakora et al. 2005; Narciso-Ortiz et al. 2020). 160 eggs were sampled during the experiment for B-esterase analysis on days 7, 14, 20, 26, 32, and 36 (Fig. 1). In each collection, embryos were classified according to the scale of Naef, (1928), which separates the three critical embryo developmental phases: blastulation (Stages I to VII), organogenesis (Stages VIII to XIV-XV), and growth (Stages XV to XX to the time of hatching). Seawater in the tanks was maintained under a constant temperature of 24 °C, a salinity of 36 PSU, and 7 mg/L of dissolved oxygen. The tanks were cleaned daily, and the water was changed on alternate days.

Individualized eggs were homogenized with 0.05 M Tris Buffer pH 7.4 at 1: 100 (w/v) each sampling time, using a Potter–Elvehjem homogenizer. After homogenization, 200 µL per sample were separated from the homogenate and centrifuged at 10,000 rpm for 5 min at 4° C. The supernatant was separated and stored at − 80 °C.

AChE activity was measured using a modified Ellman et al. (1961) method adapted to a microplate (Rodríguez-Fuentes et al. 2008). First, in each well, 10 µL of the sample were added, and 180 µL of 5, 5'-dithiobis (2 nitrobenzoic acid) (DTNB) in Tris Buffer pH 7.4, 0.05 M. This operation was carried out in duplicate. Then, the reaction was started by adding 10 µL of acetylcholine iodide (1 mM). The result of the reaction was measured at an absorbance of 405 nm for 120 s in the kinetic module.

CbE activity was measured using the method of Hosokawa and Satoh (2001). First, 10 µL of each sample was added to each well. Then, the reaction was started by adding 190 µL of Tris Buffer 7.4/ƿNPA solution. This procedure was performed in duplicate. Finally, the reaction was measured at an absorbance of 405 nm for 5 min in the kinetic module.

The protein content in the samples was determined by Bradford (1976), using bovine serum albumin (BSA) as a standard. Enzyme activities were normalized with respect to their protein content.

Phe concentrations were verified by solid-phase microextraction and gas chromatography-mass spectrometry (SPME/GC–MS). A 10 mL sample aliquot was placed in a 20 mL-SPME vial (Supelco, USA) with a PTFE/silicone septum (SU860101, Supelco). Phe extraction was performed by direct immersion for 20 min at 55 °C with a 65 μm PDMS/DVB SPME fiber (57310-U, Supelco) and magnetic stirring at 700–900 rpm. After extraction, Phe was quantified using an Agilent Technologies 7890B Series Gas Chromatography System equipped with a 5977B mass detector and a J&W HP-5MS capillary column (30 m X 0.25 mm and 0.25 μm of film thickness). The inlet temperature was 250 °C, and the samples were injected in split mode (50:1) and 10 min of desorption time. The oven temperature program started at 60 °C, then increased 10ºC/min to 250 °C (hold time 1 min); the carrier gas was helium (ultra-pure grade) with a flow rate of 0.8 mL/min. Mass spectra (m/z 50–600) were recorded at a rate of five scans per second at 70 eV, and interface and ion source temperatures were 290 and 230 °C, respectively. The MS ionization mode was electronic ionization (EI). Calibration solutions were used to quantify Phe in the samples. The detection limit of the method was 0.5 µg/L.

The data were analyzed using a multivariate approach. Permutational MANOVA (PERMANOVA) and Principal Coordinate Analysis (PCO) were performed using Primer v 7.0 + PERMANOVA add-on. Data were transformed using the function log10(x + 1) and normalized. The resemblance was calculated using the Euclidean distance of samples (Legendre 2018); PERMANOVA was done using the permutation of the residuals under a reduced model with 9999 permutations to generate the pseudo-F (Anderson 2008). Permutational multiple pair-wise tests w were used to compare the centroids of the combination of two factors, the phase of development (blastula, organogenesis, activation, and growth; four levels) and the treatment (control, solvent control, 10 and 100 µg/L Phe).

## Results and Discussion

Mean (± one standard deviation) measured concentrations of phenanthrene were 7.0 ± 0.2 and 90.0 ± 11.1 μg/L for nominal concentrations of 10 and 100 μg/L, respectively. Phenanthrene was not detected in the control and solvent-control water samples.

PCO applied to esterase activities of egg octopus exposed to Phe is presented in Fig. 1A; the first principal coordinate (horizontal) is mainly contributed to AChE activities (r = 0.93), the second principal coordinate is primarily influenced by CbE activities (*p* =  − 0.80). The graph of the centroids for the combined effect of the treatment and the developmental phase is shown in Fig. 1B. As the octopus embryo develops, the AChE and CbE activities increase in the control and solvent control treatments. On the other hand, it is possible to note that in PhE-treated embryos, AChE activities in the treatment of 10 µg/L increased at organogenesis. Still, when the embryo starts to grow, there is a marked inhibition of its activity. This same behavior is increased in embryos treated at 100 µg/L; at this concentration, AChE activities remain low and very similar to eggs at the blastula phase. CbE activities had similar behavior; the activities increase as the egg develops in the control and solvent control; on the contrary, there is an inhibitory effect that is evident at the organogenesis and growth phase.

PERMANOVA indicated a significant interaction between the factors “Phase” and “Treatment” (Pseudo F = 3.47, *p* = 0.0027), indicating that the effect of the treatments varied differently at the different phases. The post hoc paired comparisons showed no significant differences between the control and the solvent control at the different phases. Still, treatments with Phe were significantly different from controls. In addition, treatments of 10 and 100 µg/L were significantly different.

B-esterases are a family of hydrolases in all tissues and organs and are involved in the metabolism of endogenous substances or xenobiotics (Satoh and Hosokawa 1998); their inhibition is usually used as a biomarker for organophosphate exposure (Makhaeva et al. 2009). Still, it has been reported that many other pollutants could also act as inhibitors (for example, Omedes et al. 2022; Sole et al. 2022), including Phe (Richardi et al. 2018). Previous reports also indicate that B-esterases present in two cephalopods are sensitive to chemicals of environmental concern (Omedes et al. 2022).

One of the systems that first develop in *O. maya* is the nervous system (Caamal-Monsreal et al. 2016; Sanchez-García et al. 2017), which occurs in organogenesis with the appearance of the eye spot (Farías et al. 2009; Olivares et al. 2019; Uriarte and Farías 2014). AChE activity was detected at low concentrations at the blastula phase in the present study, the values increased in the controls at organogenesis. This phase culminates with the start of the circulatory system (Sanchez-García et al. 2017). Similar results of the increase of AChE were reported in zebrafish, where very low activities are at early stages until somitogenesis, where an 800% increase in AChE activity is found (Yen et al. 2011).

AChE plays an essential role in the nervous system and has also been related to cellular and physiological homeostasis (Mukherjee et al. 2016). For example, in *O. vulgaris*, it has been reported that the expression of AChE is related to cell proliferation for the regeneration of arms and that *Aplysia* sp. promotes the maturation of neural cells (Srivatsan 1999). Therefore, inhibition of this enzyme could cause detrimental effects in this organism after hatching, given its essential role in the nervous system and cell communication. Furthermore, several studies indicate that exposure to Phe induces the formation of ROS in marine invertebrates, e.g., in bivalves *Sinonovacula constricta* (Li et al. 2016) and *Pecten maximus* (Hannam et al. 2010) and oysters *Crassostrea brasiliana* (Lüchmann et al. 2014), *C. gigas* (Nogueira et al. 2017). Interestingly, it has also been reported that ROS and oxidative stress play a role in the regulation and activity of AChE (Rodríguez-Fuentes et al. 2015) and could the inhibition of AChE found in *O. maya* embryos.

CbE represents a defense system that detoxifies several substrates (Wheelock et al. 2008). Its activity seems to act differently depending on the xenobiotic, the exposure time, the organism, and even the abiotic conditions. For example, in the earthworm *Eisenia foetida* exposed to PAHs, CbE was inhibited (Nam et al. 2015), but when the same species was exposed to ash, CbE was induced (Tomazetti et al. 2017). In mammalians, exposure to environmental pollutants or lipophilic drugs can increase CbE activity (Satoh and Hosokawa 1998). Larvae of the insect *Lymantria dispar* had increased activities of CbE in polluted forests at 23 °C, but it was inhibited in higher and lower temperatures than 23 °C. The mosquito *Chironomus sancticaroli* exposed to Phe presented increased CbE activities at concentrations of 1.01 and 1.21 mg/L at 48 h but did not differ from the control at lower concentrations at 24, 72, or 96 h of exposure (Richardi et al. 2018). In the gastropod *Biomphalaria straminea* exposed to an organophosphate insecticide, there was a decrease in CbE and an increase in GST, an enzyme of phases II of biotransformation (Cossi et al. 2018), which indicates that the organism can use different metabolic strategies as a response to environmental contamination. The authors also found an increase in ROS, although they did not correlate with a decrease in CbE. On the other hand, Ortega-Ramírez (2019) related the rise in CbE activity to embryonic development in *O. maya*. She found a strong correlation between increased CbE activity and decreased yolk, indicating that CbE may play an essential role in embryonic growth and development via fat metabolism (Lian et al. 2018). So, exposure of embryos to Phe could generate less-developed individuals.

In conclusion, B-esterases in *O. maya* embryos were inhibited by Phe at all developmental phases, resulting in a promising biomarker of exposure. However, given the importance of B-esterases in many cellular functions, their inhibition may cause detrimental effects in organisms. Therefore, it is recommended that more studies be carried out to investigate the impact of pollutants in cephalopods and better understand Phe's toxicodynamics in this species.

## References

[CR1] Aguilar L, Dzul-Caamal R, Rendón-von Osten J, da Cruz AL (2022). Effects of polycyclic aromatic hydrocarbons in *Gambusia yucatana,* an Endemic Fish from Yucatán Peninsula, Mexico. Polycycl Aromat Compd.

[CR2] Anderson MJ (2008). A new method for non-parametric multivariate analysis of variance. Austral Ecol.

[CR3] Anyakora C, Ogbeche A, Palmer P, Coker H (2005). Determination of polynuclear aromatic hydrocarbons in marine samples of Siokolo Fishing Settlement. J Chromatogr A.

[CR4] Baršiene J, Lehtonen KK, Koehler A, Broeg K, Vuorinen PJ, Lang T, Pempkowiak J, Šyvokiene J, Dedonyte V, Rybakovas A, Repečka R, Vuontisjärvi H, Kopecka J (2006). Biomarker responses in flounder (Platichthys flesus) and mussel (Mytilus edulis) in the Klaipėda-Būtingė area (Baltic Sea). Mar Pollut Bull.

[CR5] Bhagat J, Sarkar A, Ingole BS (2016). DNA damage and oxidative stress in marine gastropod morula granulata exposed to phenanthrene. Water Air Soil Pollut.

[CR6] Bradford M (1976). A rapid and sensitive method for the quantitation of microgram quantities of protein utilizing the principle of protein-dye binding. Anal Biochem.

[CR7] Caamal-Monsreal C, Uriarte I, Farias A, Díaz F, Sánchez A, Re D, Rosas C (2016). Effects of temperature on embryo development and metabolism of *O. maya*. Aquaculture.

[CR8] Chen H, Zhang Z, Tian F, Zhang L, Li Y, Cai W, Jia X (2018). The effect of pH on the acute toxicity of phenanthrene in a marine microalgae *Chlorella salina*. Sci Rep.

[CR9] Coronado E, Salas S, Cepeda-González MF, Chuenpagdee R (2020). Who’s who in the value chain for the Mexican octopus fishery: mapping the production chain. Mar Policy.

[CR10] Cossi PF, Herbert LT, Yusseppone MS, Pérez AF, Kristoff G (2018). Environmental concentrations of azinphos-methyl cause different toxic effects without affecting the main target (cholinesterases) in the freshwater gastropod *Biomphalaria straminea*. Ecotoxicol Environ Saf.

[CR11] de Campos MF, de Nostro FL, da Cuña RH, Moreira RG (2018). Endocrine disruption of phenanthrene in the protogynous dusky grouper *Epinephelus marginatus* (Serranidae: Perciformes). Gen Comp Endocrinol.

[CR12] Farías A, Uriarte I, Hernández J, Pino S, Pascual C, Caamal C, Domíngues P, Rosas C (2009). How size relates to oxygen consumption, ammonia excretion, and ingestion rates in cold (Enteroctopus megalocyathus) and tropical (*Octopus maya*) octopus species. Mar Biol.

[CR13] Fu J, Sheng S, Wen T, Zhang ZM, Wang Q, Hu QX, Li QS, An SQ, Zhu HL (2011). Polycyclic aromatic hydrocarbons in surface sediments of the Jialu River. Ecotoxicology.

[CR14] Hannam ML, Bamber SD, Galloway TS, John Moody A, Jones MB (2010). Effects of the model PAH phenanthrene on immune function and oxidative stress in the haemolymph of the temperate scallop *Pecten maximus*. Chemosphere.

[CR15] Hosokawa M, Satoh T (2001). Measurement of carboxylesterase (CES) activities. Curr Protoc Toxicol.

[CR16] Isaacs J, Erikson D, Stephl M, Osowski K, Moss K (1998) Exxon Valdez oil spill restoration project final report. Exxon Valdez Oil Spill Restoration Project Final Report, REPORT 97230

[CR17] Laguerre C, Sanchez-Hernandez JC, Köhler HR, Triebskorn R, Capowiez Y, Rault M, Mazzia C (2009). B-type esterases in the snail *Xeropicta derbentina*: an enzymological analysis to evaluate their use as biomarkers of pesticide exposure. Environ Pollut.

[CR18] Legendre P (2018). Numerical ecology. Encycl Ecol.

[CR19] Li L, Jiang M, Shen X (2016). Variability in antioxidant/detoxification enzymes of Sinonovacula constricta exposed to benzo[a]pyrene and phenanthrene. Mar Pollut Bull.

[CR20] Lian J, Nelson R, Lehner R (2018). Carboxylesterases in lipid metabolism: from mouse to human. Protein Cell.

[CR21] Lüchmann KH, Mattos JJ, Siebert MN, Granucci N, Dorrington TS, Bícego MC, Taniguchi S, Sasaki ST, Daura-Jorge FG, Bainy ACD (2011). Biochemical biomarkers and hydrocarbons concentrations in the mangrove oyster *Crassostrea brasiliana* following exposure to diesel fuel water-accommodated fraction. Aquat Toxicol.

[CR22] Lüchmann KH, Dafre AL, Trevisan R, Craft JA, Meng X, Mattos JJ, Zacchi FL, Dorrington TS, Schroeder DC, Bainy ACD (2014). A light in the darkness: new biotransformation genes, antioxidant parameters and tissue-specific responses in oysters exposed to phenanthrene. Aquat Toxicol.

[CR23] Makhaeva G, Rudakova E, Boltneva N, Sigolaeva L, Eremenko A, Kurochkin I, Richardson R (2009). Blood esterases as a complex biomarker for exposure to organophosphorus compounds. NATO Sci Peace Secur Ser A Chem Bioly.

[CR24] Markaida U, Gilly WF (2016). Cephalopods of Pacific Latin America. Fish Res.

[CR25] Mukherjee S, Ray M, Ray S (2016). Shift in aggregation, ROS generation, antioxidative defense, lysozyme and acetylcholinesterase activities in the cells of an Indian freshwater sponge exposed to washing soda (sodium carbonate). Comp Biochem Physiol Part c: Toxicol Pharmacol.

[CR26] Naef A (1928). Die Cephalopoden. Embryologie. Fauna Flora Golf Neapel.

[CR27] Nam TH, Jeon HJ, Mo HH, Cho K, Ok YS, Lee SE (2015). Determination of biomarkers for polycyclic aromatic hydrocarbons (PAHs) toxicity to earthworm (Eisenia fetida). Environ Geochem Health.

[CR28] Narciso-Ortiz L, Vargas-García KA, Vázquez-Larios AL, Quiñones-Muñoz TA, Hernández-Martínez R, Lizardi-Jiménez MA (2020). Coral reefs and watersheds of the Gulf of Mexico in Veracruz: hydrocarbon pollution data and bioremediation proposal. Regional Stud Mar Sci.

[CR29] Nogueira DJ, Mattos JJ, Dybas PR, Flores-Nunes F, Sasaki ST, Taniguchi S, Schmidt ÉC, Bouzon ZL, Bícego MC, Melo CMR, Toledo-Silva G, Bainy ACD (2017). Effects of phenanthrene on early development of the Pacific oyster *Crassostrea gigas* (Thunberg, 1789). Aquat Toxicol.

[CR30] Olivares A, Rodríguez-Fuentes G, Mascaró M, Arteaga AS, Ortega K, Monsreal CC, Tremblay N, Rosas C (2019). Maturation trade-offs in octopus females and their progeny: energy, digestion and defence indicators. PeerJ.

[CR31] Omedes S, Andrade M, Escolar O, Villanueva R, Freitas R, Solé M (2022). B-esterases characterisation in the digestive tract of the common octopus and the European cuttlefish and their in vitro responses to contaminants of environmental concern. Environ Res.

[CR32] Ortega-Ramírez KM (2019) Evaluación de biomarcadores bioquímicos de balance redox durante el desarrollo embrionario de *Octopus maya* (Voss & Solís-Ramírez, 1966). Tesis de maestría. Universidad Nacional Autónoma de México, Sisal. Yucatán, Mexico

[CR33] Pascual C, Cruz-Lopez H, Mascaró M, Gallardo P, Sánchez A, Domingues P, Rosas C (2020). Changes in biochemical composition and energy reserves associated with sexual maturation of *Octopus maya*. Front Physiol.

[CR34] Richardi VS, Vicentini M, Morais GS, Rebechi D, da Silva TA, Fávaro LF, Navarro-Silva MA (2018). Effects of phenanthrene on different levels of biological organization in larvae of the sediment-dwelling invertebrate *Chironomus sancticaroli* (Diptera: Chironomidae). Environ Pollut.

[CR35] Rico EP, Rosemberg DB, Dias RD, Bogo MR, Bonan CD (2007). Ethanol alters acetylcholinesterase activity and gene expression in Zebrafish brain. Toxicol Lett.

[CR36] Rodríguez-Fuentes G, Armstrong J, Schlenk D (2008). Characterization of muscle cholinesterases from two demersal flatfish collected near a municipal wastewater outfall in Southern California. Ecotoxicol Environ Saf.

[CR37] Rodríguez-Fuentes G, Rubio-Escalante FJ, Noreña-Barroso E, Escalante-Herrera KS, Schlenk D (2015). Impacts of oxidative stress on acetylcholinesterase transcription, and activity in embryos of zebrafish (Danio rerio) following Chlorpyrifos exposure. Comp Biochem Physiol Part C Toxicol Pharmacol.

[CR38] Rodríguez-Fuentes G, Marín-López V, Hernández-Márquez E (2016). Cholinesterases in *Gambusia yucatana*: biochemical characterization and its relationship with sex and total length. Bull Environ Contam Toxicol.

[CR39] Rosas C, Gallardo P, Mascaró M, Caamal-Monsreal C, Pascual C, Iglesias J, Fuentes L, Villanueva R (2014). Octopus maya. Cephalopod culture.

[CR40] Sanchez-García A, Rodríguez-Fuentes G, Díaz F, Galindo-Sánchez CE, Ortega K, Mascaró M, López E, Caamal-Monsreal C, Juárez O, Noreña-Barroso E, Re D, Rosas C (2017). Thermal sensitivity of *O. maya* embryos as a tool for monitoring the effects of environmental warming in the Southern of Gulf of Mexico. Ecol Indicators.

[CR41] Satoh T, Hosokawa M (1998). The mammalian carboxylesterases: from molecules to functions. Annu Rev Pharmacol Toxicol.

[CR42] Sauer WHH, Gleadall IG, Downey-Breedt N, Doubleday Z, Gillespie G, Haimovici M, Ibáñez CM, Katugin ON, Leporati S, Lipinski MR, Markaida U, Ramos JE, Rosa R, Villanueva R, Arguelles J, Briceño FA, Carrasco SA, Che LJ, Chen CS (2021). World octopus fisheries. Rev Fish Sci Aquac.

[CR43] Sole M, Figueres E, Mañanós E, Rojo-Solís C, García D (2022). Characterisation of plasmatic B-esterases in bottlenose dolphins (Tursiops Truncatus) and their potential as biomarkers of plastic-related chemical exposures. SSRN Electron J.

[CR44] Srivatsan M (1999). Effects of organophosphates on cholinesterase activity and neurite regeneration in Aplysia. Chem Biol Interact.

[CR45] Tran TKA, Yu RMK, Islam R, Nguyen THT, Bui TLH, Kong RYC, O’Connor WA, Leusch FDL, Andrew-Priestley M, MacFarlane GR (2019). The utility of vitellogenin as a biomarker of estrogenic endocrine disrupting chemicals in molluscs. Environ Pollut.

[CR46] Turja R, Sanni S, Stankevičiūtė M, Butrimavičienė L, Devier MH, Budzinski H, Lehtonen KK (2020). Biomarker responses and accumulation of polycyclic aromatic hydrocarbons in *Mytilus trossulus* and *Gammarus oceanicus* during exposure to crude oil. Environ Sci Pollut Res.

[CR47] Uriarte Í, Farías A, Iglesias J, Fuentes L (2014). Robsonella fontaniana. Cephalopod culture.

[CR48] Vieira LR, Sousa A, Frasco MF, Lima I, Morgado F, Guilhermino L (2008). Acute effects of Benzo[a]pyrene, anthracene and a fuel oil on biomarkers of the common goby Pomatoschistus microps (Teleostei, Gobiidae). Sci Total Environ.

[CR49] Voss GL, Solís-Ramírez M (1966). Octopus maya, a new species from The Bay of Campeche, Mexico. Bull Mar Sci.

[CR50] Wheelock CE, Phillips BM, Anderson BS, Miller JL, Miller MJ, Hammock BD, Whitacre DM (2008). Applications of carboxylesterase activity in environmental monitoring and toxicity identification evaluations (TIEs). Reviews of environmental contamination and toxicology.

[CR51] Yen J, Donerly S, Levin ED, Linney EA (2011). Differential acetylcholinesterase inhibition of chlorpyrifos, diazinon and parathion in larval zebrafish. Neurotoxicol Teratol.

